# School attendance in individuals with cerebral palsy in Brazil

**DOI:** 10.1111/dmcn.70316

**Published:** 2026-05-21

**Authors:** Ricardo Rodrigues de Sousa Junior, Luana Cristina da Silva, Ana Cristina Resende Camargos, Eliane Beatriz Cunha Policiano, Lívia Ferreira Coutinho Alonso, Nadia Badawi, Israt Jahan, Gulam Khandaker, Maria De Las Mercedes Ruiz Brunner, Paula Silva de Carvalho Chagas, Hércules Ribeiro Leite

**Affiliations:** ^1^ Graduate Program in Rehabilitation Sciences, School of Physical Education, Physical Therapy and Occupational Therapy Universidade Federal de Minas Gerais Belo Horizonte Brazil; ^2^ Brazilian Register of Cerebral Palsy Patient and Involvement Group, School of Physical Education, Physical Therapy and Occupational Therapy Universidade Federal de Minas Gerais Belo Horizonte Brazil; ^3^ Cerebral Palsy Alliance Research Institute, Specialty of Child & Adolescent Health, Sydney Medical School, Faculty of Medicine & Health The University of Sydney Sydney New South Wales Australia; ^4^ School of Health, Medical and Applied Sciences Central Queensland University Rockhampton Queensland Australia; ^5^ Central Queensland Hospital and Health Service Rockhampton Queensland Australia; ^6^ Instituto Universitario de Ciencias Biomédicas de Córdoba (IUCBC) Centro de Investigación en Medicina Traslacional ‘Severo R. Amuchástegui’ (CIMETSA), Consejo Nacional de Investigaciones Científicas y Técnicas (CONICET) Córdoba Argentina; ^7^ Graduate Program in Rehabilitation Sciences and Physical‐Functional Performance, Faculty of Physical Therapy Universidade Federal de Juiz de Fora Juiz de Fora Brazil; ^8^ CanChild McMaster University Hamilton Ontario Canada

## Abstract

**Aim:**

To identify factors associated with school attendance among Brazilian children, adolescents, and young adults with cerebral palsy (CP) using population‐based data from the Brazilian Cerebral Palsy Register (BrCPR).

**Method:**

This cross‐sectional study analysed data from 774 individuals aged 4 to 21 years with CP registered in the BrCPR. Variables included sociodemographic and clinical characteristics (Gross Motor Function Classification System, Manual Ability Classification System, and speech limitations), and school attendance. Descriptive and bivariate analyses were conducted using *χ* ^2^ tests; multivariable logistic regression models were applied to estimate adjusted odds ratios (aORs) with corresponding 95% confidence intervals (CIs).

**Results:**

Among participants, 76% attended school, although 62% could not read and write. Adolescents with CP aged 13 to 21 years were less likely to attend school compared to those aged 4 to 12 years (aOR = 0.49, 95% CI = 0.3–0.7), as were those classified in Gross Motor Function Classification System levels IV and V compared to levels I to III (aOR = 0.29, 95% CI = 0.1–0.4). Additionally, the presence of speech limitations was associated with reduced school attendance (aOR = 0.12, 95% CI = 0.1–0.3).

**Interpretation:**

Although school attendance rates for Brazilian individuals with CP are relatively high compared to other low‐ and middle‐income countries, major barriers persist. Addressing speech support, and mobility‐related inclusion, is essential to improve both school attendance and meaningful involvement.

## Graphical abstract

This graphical abstract summarizes the main findings of the study.

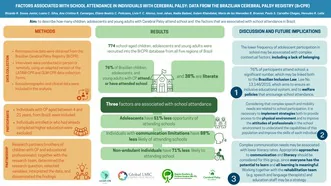

Portuguese translation of this Original Article is available in the online issue.

**Abbreviations**
BrCPR: Brazilian Cerebral Palsy Register
LMIC: low‐ and middle‐income country
PPI: patient and public involvement

**What this paper adds**

- Seventy‐six per cent of individuals with cerebral palsy in Brazil attend school.
- Non‐attendance in school was linked to older age.
- Non‐attendance in school was associated with limitations in motor function and speech.

Participation can be defined as involvement and attendance in real‐life activities.^1^ When children engage in enjoyable activities across different settings, their health and well‐being improve.^2^ However, in Brazil, a low‐ and middle‐income country (LMIC), recent data have shown low levels of participation among children with disabilities, such as those with cerebral palsy (CP), across different settings. This participation tends to decrease with increasing age and with greater mobility limitations.^3^ According to the updated definition of CP by Dan et al.,^4^ this population ‘… frequently encounter primary and secondary impairments across various areas of development and functioning, which can significantly impact their participation in daily life …’.

School attendance (here meaning physically being present in school) marks an important expansion of the child's social environment and supports the development of academic and everyday life skills.^5^ One of the United Nations Sustainable Development Goals is that by 2030, ‘all girls and boys complete free, equitable and quality primary and secondary education leading to relevant and effective learning outcomes’.^5^ Children with CP may encounter restrictions in school participation, relative to the absence of proper teacher training, lack of full educational support, physical and attitudinal barriers, and bullying and harassment.^6^ Despite the establishment of educational policies of promoting the inclusion of children with disabilities across all school settings, and the obligation to comply with the United Nations Convention on the Rights of the Child and the Convention on the Rights of Persons with Disabilities, many barriers to full participation persist.^7^ For instance, people with disabilities have lower school attendance, school completion, years of schooling, and literacy levels.^6^ These challenges are even more pronounced in LMICs.

A recent study presenting data on school attendance among children with CP in Bangladesh revealed that, although 1582 children were of school age, only 26.1% had attended school.^6^ Data from other LMICs presented similar results concerning school attendance by children with CP: in Sudan, 12.3%;^8^ in Benin, 23.3%;^9^ in Uganda, 30.0%;^10^ and in India, 40.0%.^11^ Adolescents with CP, individuals with bilateral CP, and speech impairments were presented with more barriers to attending school in Bangladesh.^6^ Children with CP with a higher frequency of comorbidities, with learning difficulties, visual acuity impairment, and epilepsy were more likely to have lower school attendance in Nigeria.^12^ The severity of gross motor and manual functioning was also associated with decreased odds of school attendance.^6^, ^12^

At the beginning of the century, Brazil did not have an educational system plan for the inclusion of people with disabilities, despite the 1988 Constitution guaranteeing access to education without discrimination.^13^ This scenario began to change with the strengthening forces in favour of inclusion by the Salamanca Declaration and the Guatemala Convention.^14^ The Brazilian Law for the Inclusion of Persons with Disabilities, which includes the right for children with disabilities to attend all educational systems, is completing 10 years.^15^ This was a great achievement for a country with 11 million people unable to read or write. Of the 151 million people who go to school, 47.4% did not complete secondary education.^16^ In Brazil, 22.3% of people with disabilities are not literate. This number is four times higher than in the typically developing Brazilian population.^16^ Data from children with CP attending school in Brazil are rare. The PartiCipa Brazil preliminary study showed that one of four school‐age individuals with CP were out of school.^17^

Initiatives to better understand the epidemiology, inclusion, and participation of children with CP in LMICs have emerged, such as the Global Low‐ and Middle‐Income Countries Cerebral Palsy Registry.^18^ In Latin America specifically, the Latin American Cerebral Palsy Register is collecting data from several countries across the region, with Brazil being the most populous and largest participating country.^19^ The Brazilian Cerebral Palsy Register (BrCPR) began data collection in September 2024.^20^ The aim of this study was to describe school attendance among individuals with CP in Brazil and identify factors associated with school attendance. This information is important for guiding public policies and investments in advocacy and school inclusion for individuals with CP in Brazil.

## METHOD

Retrospective data from the BrCPR were used in this study to report on school attendance of children, adolescents, and young adults with CP in Brazil.

### Brazilian Cerebral Palsy Register

The BrCPR is a national surveillance system established in 2024 through a collaboration between researchers, practitioners, international partners, and an active patient and public involvement (PPI) group. It integrates global efforts such as the Global Low‐ and Middle‐Income Countries Cerebral Palsy Registry and Latin American Cerebral Palsy Register to expand knowledge on the epidemiology of CP in LMICs.^19^ The BrCPR adopts the International Classification of Functioning, Disability and Health as its organizing framework, enabling a more comprehensive understanding of human functioning by incorporating activities, participation, body functions and structures, and personal and environmental factors. The registry uses both in‐person (e.g. hospitals, clinics, and health centres) and remote data collection strategies across all five regions of Brazil, in urban and rural cities. The BrCPR registers individuals with CP from 0 to 100 years old. The PPI group, including adults with CP, mothers of children with CP, practitioners, and researchers, has been involved since the registry's inception, contributing to variable selection and dissemination materials to ensure that the perspectives of individuals with CP guide project priorities.^20^

### Study participants, inclusion/exclusion criteria and data collection

Study participants were individuals registered with the BrCPR. For this study, we retrieved data from the BrCPR of participants aged between 4 years and 21 years. Mandatory school age in Brazil is 4 to 17 years. In this study, we extended the age range up to 21 years to also include those enrolled in the Brazilian young people and adult education programmes. Therefore, for this study, between August of 2024 and May of 2025, 774 children and young people were recruited into the BrCPR database from all five regions of Brazil.

Detailed information on registering individuals with confirmed CP in the BrCPR has been published previously by our team.^20^ Data were collected by interviewing the individual with CP or their primary caregivers (in person at clinics, rehabilitation centres, hospitals, and health centres, and remotely). Each interview lasted a maximum of 30 minutes. All researchers received training from the BrCPR team regarding data collection. The data collection form was adapted from the version recommended by the Latin American Cerebral Palsy Register and the Global Low‐ and Middle‐Income Countries Cerebral Palsy Registry and was administered using an online platform (REDCap, Vanderbilt University, Nashville, TN, USA). This form is available as Appendix S1.

### Data management

Selected sociodemographic and clinical variables were retrieved from the BrCPR database. The following sociodemographic characteristics were retrieved: participant's age, sex, Brazilian region and state where living, school attendance (if participants physically attended school), school level (primary school, including early childhood education, elementary and middle school, mostly known as elementary school in Brazil; and secondary school, mostly known as high school), literacy (ability to read or write), rehabilitation attendance, use of assistive devices, information of who was the family primary earner, household monthly income, educational level of main parent or caregiver, and access to public and private health care. This information was obtained according to the answers provided by parents or caregivers during the BrCPR interviews.

Clinical characteristics, such as level of mobility (Gross Motor Function Classification System [GMFCS]), level of manual ability (Manual Ability Classification System [MACS]), CP motor type (spastic, dyskinetic, ataxic, hypotonic, or mixed), CP topography (unilateral or bilateral) for spastic CP, associated impairments (intellectual or speech), and the presence of comorbidities (hip dislocation, epilepsy, reflux, hearing problems, visual impairments, and swallowing difficulties) were collected. All clinical characteristics were collected according to parent and caregiver perceptions, and confirmed in medical charts and by clinicians, when available. We used the GMFCS Family Report version and the MACS interview flow chart to accurately collect parent and caregiver perceptions based on participants' performance.^21^

### Statistical analysis

Descriptive statistics (frequencies or means with SDs) were first used to summarize participants' demographic, clinical, and contextual characteristics, including age, sex, rehabilitation attendance, Brazilian region and state, assistive device use, literacy (i.e. the ability to read and write), access to health care, CP type, CP topography, GMFCS level, and MACS level.

To explore the initial associations between each independent variable and school attendance, we performed bivariate analyses using the *χ* ^2^ test. Using bivariate screening, school attendance (yes/no) was defined a priori as the dependent variable. All sociodemographic and clinical variables that were considered as candidate factors were based on clinical relevance, theoretical plausibility, and evidence from previous CP epidemiological studies. These included: sex (female/male); age group (4–12 years and 13–21 years); school level (primary/secondary); literacy (yes/no); primary earner (mother or father/others); household income (below vs above two Brazilian minimum monthly income: < R$3000/US$538); parental or caregiver education (incomplete secondary or lower vs complete secondary or higher); motor type (spastic/other); topography (unilateral/bilateral); GMFCS level (ambulant: GMFCS levels I–III; non‐ambulant: GMFCS levels IV and V); MACS level (independent hand use: MACS levels I and II; non‐independent hand use, i.e. needing adaptations or caregiver support: MACS levels III–V); intellectual disability (yes/no); visual limitations (yes/no); speech limitations (yes/no); and comorbidities (none vs ≥1).

Logistic regression models were first fitted to estimate crude associations between each variable and school attendance. To avoid model overfitting and ensure parsimony, only variables that met two criteria were entered into the adjusted multivariate logistic regression model: (1) statistically significant in *χ* ^2^ bivariate analyses (*p* < 0.05); and (2) conceptually justified based on prior literature on school participation in children with CP.

Before fitting the adjusted model, we assessed multicollinearity among all candidate factors using the standard variance inflation factor. All variance inflation factor values were below 3, indicating that multicollinearity was not a concern.^22^, ^23^ In our adjusted model, statistically significant variables in the unadjusted model were included. Final models were reported as odds ratios (ORs) and adjusted ORs (aOR) with 95% confidence intervals (CIs).

Missing data were examined for patterns; a complete‐case approach was adopted given the low proportion of missingness and the absence of evidence for systematic bias. All analyses were performed using SPSS v26 (IBM Corp., Armonk, NY, USA).

### Patient and public involvement

The BrCPR uses the PPI methodology. This group includes four researchers, six mothers of children with CP (*n* = 1 GMFCS level II; *n* = 2 GMFCS level III; *n* = 3 GMFCS level V), and two adults with CP (GMFCS level IV). Their roles within the registry were defined based on the PPI matrix of involvement framework.^24^ For the present study, two mothers of the main group were invited. Their roles were to be co‐thinkers and partners in research. The PPI group has been involved since the inception of the BrCPR and participated in the process of translating and adapting the data collection form, suggesting the addition of relevant questions and modifications to the response options for certain items. In the present study, members defined the research question that guided the study based on discussion of parents' difficulties in their children's school participation. They also assisted in determining the variables to be included in the analyses, and contributed to the interpretation of the results and the dissemination of findings through the design of an infographic.

### Ethical considerations

The study was approved by the ethics committee of the Universidade Federal de Minas Gerais (approval no. 79576124.2.0000.5149). All caregivers of individuals with CP or the individuals themselves signed the informed consent form. Additionally, children and adolescents capable of understanding the study's objectives also signed an assent form.

## RESULTS

### Characteristics of study participants

The mean age at registration was 9 years 9 months (SD = 4 years 3 months) and 331 (42.8%) were female. Figure 1 presents the proportion of participants in each region and state of Brazil. The main characteristics of this group are presented in Table 1. Among this group, 589 (76%) attended school and 485 (62%) were not literate; 74% of those illiterate individuals were aged 6 years or older (i.e. the full expected literacy age in Brazil). Among all participants, 567 (73.3%) had spastic CP, 429 (55.4%) were classified in GMFCS levels III to V, 580 (74.9%) had speech impairments, and 738 (95%) received any type of rehabilitation at the time of participating in the BrCPR interview. More details are shown in Tables 2 and 3.

**FIGURE 1:**
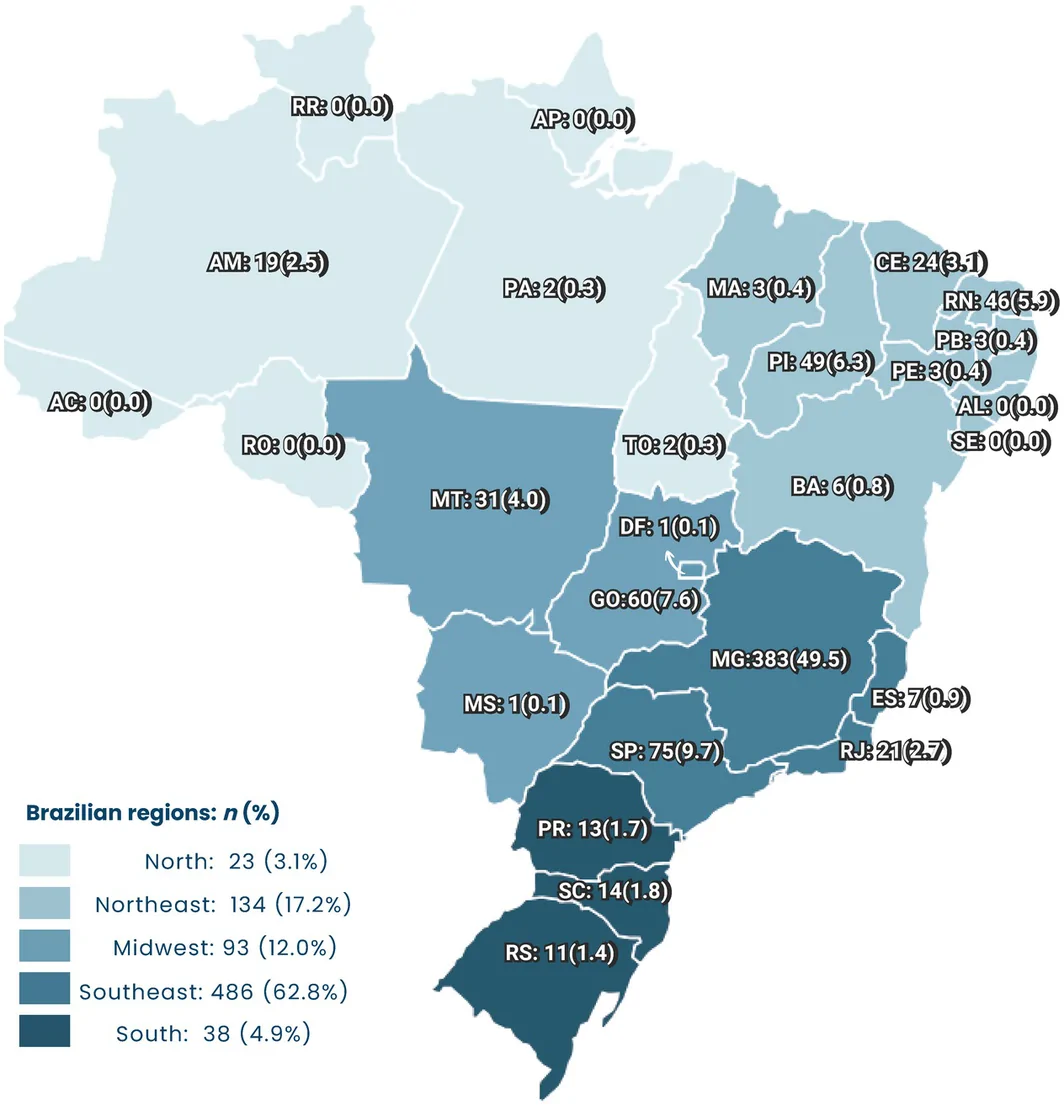
Proportion of the sample among Brazilian regions and states. AC, Acre; AL, Alagoas; AM, Amazonas; AP, Amapá; BA, Bahia; CE, Ceará; DF, Distrito Federal; ES, Espírito Santo; GO, Goiás; MA, Maranhão; MG, Minas Gerais; MS, Mato Grosso do Sul; MT, Mato Grosso; PA, Pará; PB, Paraíba; PE, Pernambuco; PI, Piauí; PR, Paraná; RJ, Rio de Janeiro; RN, Rio Grande do Norte; RO, Rondônia; RR, Roraima; RS, Rio Grande do Sul; SC, Santa Catarina; SE, Sergipe; SP, São Paulo; TO, Tocantins.

**TABLE 1:** Main characteristics of the BrCPR participants included in the study (*n* = 774).

Characteristic	*n* (%)	
Age, median (interquartile range)	9 (7)	
Sex		
Female	331 (42.8)	
Male	443 (57.2)	
GMFCS level		
I	74 (9.6)	
II	130 (16.8)	
III	112 (14.5)	
IV	149 (19.3)	
V	280 (36.2)	
MACS level		
I	102 (13.2)	
II	179 (23.1)	
III	107 (13.8)	
IV	129 (16.7)	
V	218 (28.2)	
CP type		
Spastic	592 (76.5)	
Ataxic	12 (1.6)	
Dyskinetic	61 (7.9)	
Hypotonic	39 (5)	
CP topography		
Unilateral	487 (62.9)	
Bilateral	90 (11.6)	
Access to health care services		
Public health care	630 (81.4)	
Private health care	364 (47.0)	
Rehabilitation support		
Yes	743 (96)	
No	53 (6.8)	
Have all necessary assistive devices		
Yes	391 (50.5)	
No	170 (22.0)	
Partially	182 (23.5)	
Literacy (years)	**Yes**	**No**
4–6	53 (29.9)	124 (70.1)
7–12	78 (27.6)	204 (72.4)
13–21	79 (33.4)	157 (66.6)
Total	210 (27.1)	485 (62.2)

*Note*: Missing data: GMFCS level *n* = 29 (3.7%); MACS level *n* = 39 (5%); CP type *n =* 70 (9%); topography *n* = 197 (25%); access to health care services *n* = 18 (2.3%); rehabilitation support *n* = 22 (2.8%); assistive devices *n* = 31 (4%); literacy *n* = 79 (10.7%).

Abbreviations: BrCPR, Brazilian Cerebral Palsy Register; GMFCS, Gross Motor Function Classification System; MACS, Manual Ability Classification System.

**TABLE 2:** Contextual factors associated with school attendance among children and young people with cerebral palsy in the BrCPR.

Factor	Attending school					
	Total (%)^a^	No (%)	Yes (%)		Unadjusted odds ratio (95% CI)^b^	*p*
Age (years)				0.002	Ref.	
4–12	567 (73.3)	120 (21.1)	447 (78.8)			
13–21	207 (26.7)	56 (27.1)	142 (68.6)		**0.57 (0.3–0.9)**	**0.03**
Sex				0.57	Ref.	
Female	331 (42.8)	69 (20.8)	249 (75.2)			
Male	443 (57.2)	85 (19.2)	340 (76.7)		0.90 (0.3–1.2)	0.57
School level^c^				0.17	Ref.	
Primary school	435 (56)	40 (9.2)	395 (90.8)			
Secondary school	61 (7.9)	9 (14.8)	52 (85.2)		0.69 (0.4–1.0)	0.52
Literacy				0.001	Ref.	
Yes	210 (27.1)	15 (7.1)	195 (92.2)			
No	485 (62.7)	125 (25.8)	358 (73.8)		0.58 (0.2–1.1)	0.16
Primary earner				0.20	Ref.	
Mother or father	574 (74.2)	104 (18.1)	447 (77.9)			
Other	196 (25.3)	50 (25.5)	139 (70.9)		0.64 (0.4–1.9)	0.20
Parent or caregiver education level				0.51		
Incomplete secondary school or below	321 (41.5)	73 (22.7)	233 (72.6)		Ref.	
Secondary school or higher education	428 (55.3)	74 (17.3)	339 (79.2)		1.44 (0.9–2.2)	0.60
Household income in Brazilian reais (R$) (~US$)^d^				0.20	Ref.	
< R$3000 (< US$538)	323 (41.7)	71 (22.0)	238 (73.7)			
> R$3000 (> US$538)	412 (52.2)	77 (18.7)	327 (79.4)		1.26 (0.8–1.8)	0.20

*Note*: Bold indicates statistically significant associations (*p* < 0.05).

Abbreviations: BrCPR, Brazilian Cerebral Palsy Register; CI, confidence interval.

^a^ Missing data: school level *n* = 278 (35%); literacy *n* = 79 (10.2%); primary earner *n* = 4 (0.51%); family income *n* = 39 (5.0%).

^b^ Variables included in the final model are highlighted in bold (i.e. those significant in the bivariate analysis).

^c^ Primary school: early childhood education, elementary, and middle school. Secondary school: high school.

^d^ US$1 ~ R$5.57.

**TABLE 3:** Clinical factors associated with school attendance among children and young people with cerebral palsy in the BrCPR.

Factor	Attending school					
	Total (%)^a^	No (%)	Yes (%)	*χ* ^*2*^ test significance	Unadjusted odds ratio (95% CI)^b^	*p*
CP type				0.22	Ref.	
Spastic	567 (73.3)	16 (20.5)	447 (78.8)			
Non‐spastic	105 (13.6)	88 (15.2)	116 (83.8)		1.40 (0.8–0.2)	0.22
Topography				0.001	Ref.	
Bilateral	477 (62.9)	120 (24.6)	363 (74.5)			
Unilateral	90 (11.6)	2 (2.2)	88 (97.8)		4.5 (0.5–36.5)	0.15
GMFCS level				0.001	Ref.	
Ambulant (levels I–III)	316 (40.8)	26 (8.2)	287 (90.8)			
Non‐ambulant (levels IV and V)	429 (55.4)	127 (29.6)	298 (69.5)		**0.36 (0.1–0.7)**	**0.01**
MACS level				0.001	Ref.	
I and II	282 (36.4)	20 (7.1)	260 (92.2)			
III–V	454 (58.7)	129 (28.4)	321 (70.7)		0.55 (0.2–1.7)	0.13
Intellectual impairment				0.001	Ref.	
No	199 (25.7)	23 (11.6)	176 (88.4)			
Yes	464 (59.9)	109 (23.5)	350 (75.4)		0.70 (0.3–1.3)	0.30
Speech impairment				0.001	Ref.	
No	158 (20.4)	4 (2.5)	153 (20.4)			
Yes	580 (74.9)	146 (25.2)	430 (74.1)		**0.20 (0.4–0.9)**	**0.03**
Comorbidities				0.001	Ref.	
None	129 (16.7)	8 (6.2)	115 (16.7)			
One or more	627 (81)	146 (23.3)	474 (75.6)		0.55 (0.1–1.7)	0.30

*Note*: Bold indicates statistically significant associations (*p* < 0.05).

Abbreviations: BrCPR, Brazilian Cerebral Palsy Register; CI, confidence interval; GMFCS, Gross Motor Function Classification System; MACS, Manual Ability Classification System.

^a^ Missing data: CP type *n* = 102 (13.1%); topography *n* = 207 (25.4%); GMFCS level *n* = 29 (3.7%); MACS level *n* = 38 (4.9%); intellectual impairment *n* = 111 (14.3%); speech limitations *n* = 36 (4.6%); comorbidities *n* = 18 (2.3%).

^b^ Variables included in the final model are highlighted in bold (i.e. those significant in the bivariate analysis).

### Factors related to school attendance

#### Sociodemographic factors

Among our independent variables, age and literacy were the only sociodemographic variables that were statistically significantly associated with school attendance (*p* < 0.01) attendance in our preliminary bivariate analysis (*χ* ^2^ test) (Table 2).

School attendance was higher in children aged 4 to 12 years compared with adolescents and young adults aged 13 to 21 years. Adolescents and young adults presented significantly higher unadjusted odds of not attending school (unadjusted OR = 0.57 [0.3–0.9]; *p* = 0.01). Literacy was not significantly associated with school attendance (*p* = 0.16). These results are detailed in Table 2.

#### Clinical factors

The clinical features of CP topography, GMFCS level, MACS level, presence of intellectual deficiency, speech limitations, and presence of other comorbidities showed statistically significant differences (*p* < 0.002) in school attendance in our preliminary bivariate analysis (Table 3).

Ambulant individuals with CP (those classified in GMFCS levels I–III) had higher school attendance compared with non‐ambulant individuals (those classified in GMFCS levels IV and V). Non‐ambulant individuals presented significant lower unadjusted odds of attending school (unadjusted OR = 0.36 [0.1–0.7], *p* = 0.02) compared with individuals in GMFCS levels I to III. Similarly, individuals with speech limitations were less likely to attend school than those with no limitations (unadjusted OR = 0.20 [0.4–0.9], *p* = 0.04) (Table 3). Other clinical features, such as MACS levels, the presence of intellectual disability, visual impairments, or other comorbidities, were not significantly associated with school attendance (*p* > 0.05).

### Factors associated with school attendance among individuals with CP

Variables that were significantly related to school attendance of participants in the unadjusted analysis were subsequently included in the multivariable analysis. All included variables were deemed acceptable for inclusion in our model as multicollinearity was not a concern (variance inflation factor <3).

Age, GMFCS level, and speech impairment were all significantly associated with school attendance of Brazilian individuals with CP in our adjusted regression model (Table 4). The aOR for attending school was 0.49 (95% CI = 0.3–0.7; *p* = 0.001) among adolescents and young adults with CP. This means that adolescents and young adults are more likely to not attend school than children. The aOR was 0.29 (95% CI = 0.1–0.4; *p* = 0.001) among those classified in GMFCS levels IV and V; this means that non‐ambulant individuals are less likely to attend school compared to ambulant children. The aOR was 0.12 (95% CI = 0.1–0.3, *p* = 0.002) among those with speech impairments, meaning that individuals with speech impairments are less likely to attend school compared with individuals who do not have communication limitations.

**TABLE 4:** Factors associated with school attendance among children and young people with cerebral palsy in the BrCPR.

Factor	Adjusted odds ratio (95% CI)	*p*
Age (years)		
4–12	Ref.	
13–21	0.49 (0.3–0.7)	0.001
GMFCS level		
Ambulant (levels I–III)	Ref.	
Non‐ambulant (levels IV and V)	0.29 (0.1–0.4)	0.001
Speech limitations		
No	Ref.	
Yes	0.12 (0.1–0.3)	0.001

Abbreviations: BrCPR, Brazilian Cerebral Palsy Register; CI, confidence interval; GMFCS, Gross Motor Function Classification System.

### Interpretation of the results and contributions of the PPI group

Figure 2 presents the results in plain language, as designed by the study's PPI group. This figure was created to facilitate knowledge translation among the interested parts and include insights to better promote school attendance among individuals with CP. Based on the reported results, the PPI group joined with researchers and contributed with important reflections that might explain the reported results. The ‘Discussion’ section is based on their thoughts and what they considered to be important to discuss, according to our findings.

**FIGURE 2:**
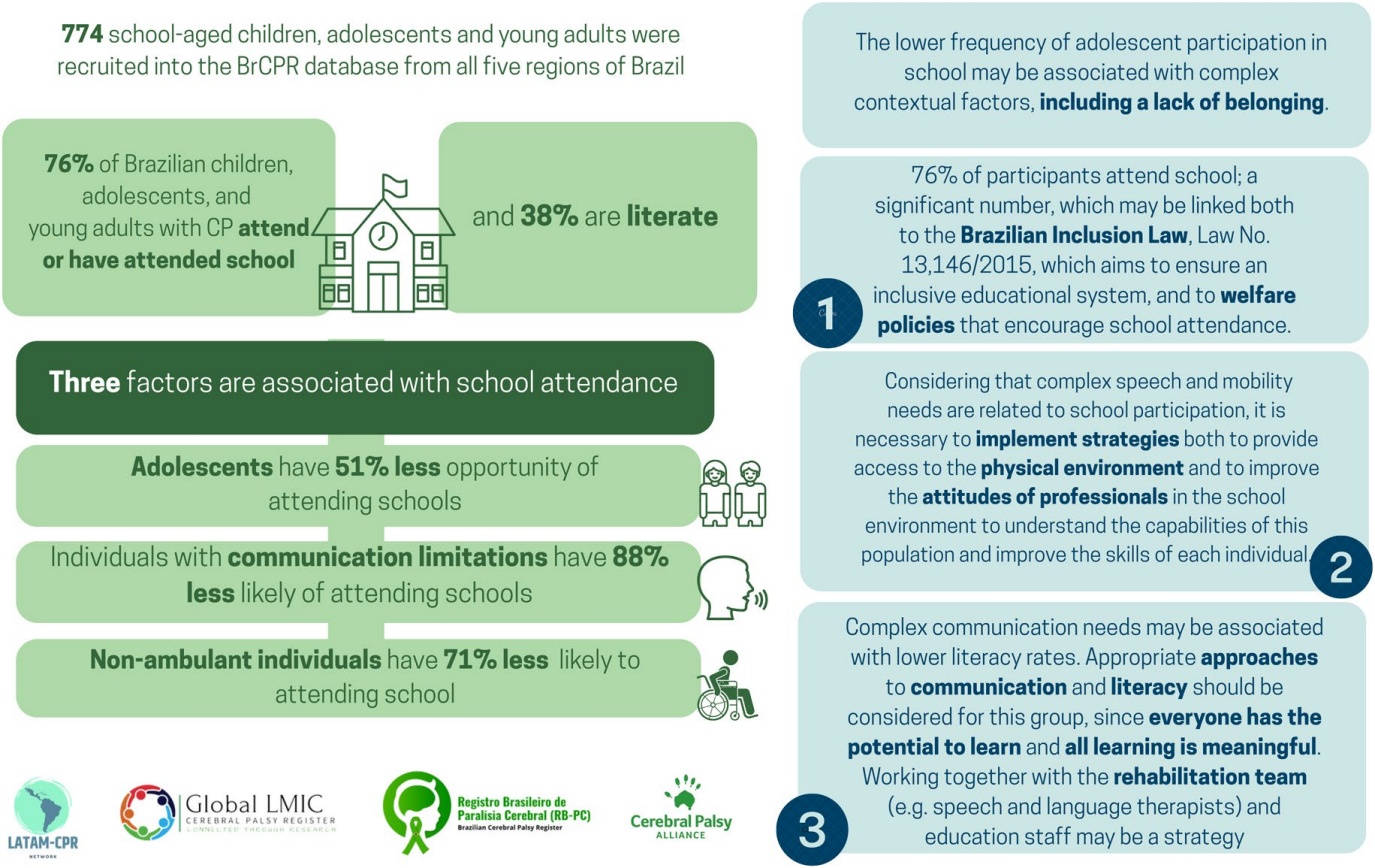
Infographic of the study results.

## DISCUSSION

The main goal of this study was to determine factors associated with school attendance among Brazilian individuals with CP. To the best of our knowledge, this is the first study that evaluated factors associated with school attendance in these individuals using population‐based data. We found that although most Brazilian people with CP attend school, key factors such as age, motor function level, and speech limitations are strongly associated with their school attendance.

According to this study, 76% of individuals with CP attend school—a positive finding given that attendance rates in other LMICs are much lower, often between <12% and 40%.^6^, ^8^, ^9^, ^10^, ^11^ These results highlight the impact of the Brazilian Law for the Inclusion of Persons with Disabilities, the National Policy on Special Education, and the Statute of the Person with Disability (Law 13.146/2015), which guarantee equal educational opportunities, access to nearby regular schools, and prohibit exclusion or neglect.^15^, ^25^, ^26^ These policies agree with international statements regarding school and education of individuals with disabilities, such as the CP Manifesto: ‘students with cerebral palsy must not be excluded from the regular education system’.^27^, ^28^ The successful implementation of these policies may help explain the higher attendance rates among Brazilian individuals with CP. However, despite this strong legal framework, gaps in school participation persist, particularly among those with more severe disabilities.

It is important to emphasize that attendance, that is, simply being present, is only one component of the broader concept of participation. Participation in life situations involves the interaction of multiple factors. According to Imms et al.,^1^ two key constructs define participation: attendance and involvement, with involvement referring to the subjective experience of participation while attending. Both constructs are shaped by various intrinsic and extrinsic factors. While attendance is a necessary and important aspect of school participation, it is not sufficient to ensure meaningful involvement. Although this study did not directly measure school involvement, the finding that 62% of school‐attending individuals with CP are not literate may suggest limited involvement in school activities. This lack of involvement may, in turn, negatively affect continued school attendance as literate children are more likely to remain in school. These results reinforce the notion that attendance and involvement are mutually reinforcing and that greater efforts are needed to enhance the quality of the academic experience for Brazilian individuals with CP.

Speech limitations were associated with school non‐attendance in individuals with CP. In the general population, children with speech impairments often face challenges at school, not only in terms of academic performance but also in overall engagement with learning.^29^ The use of augmentative and alternative communication can be a powerful strategy to support those with more severe speech limitations in the school environment. However, several barriers, such as inconsistent access to augmentative and alternative communication and a lack of teacher or staff knowledge and training, may hinder its effective use in Brazilian schools.^30^ To prevent school absenteeism in students with speech limitations, it is essential to address these barriers, which will promote inclusive education and ensure equal learning opportunities. One possible strategy to reduce these barriers is the active participation of health professionals in school settings helping students' inclusion and staff training.

Non‐ambulant individuals with CP in Brazil have lower odds of attending school, a pattern also observed in other LMICs such as Bangladesh, Sudan, India, Uganda, and Benin.^6^, ^8^, ^9^, ^10^, ^11^ This reduced attendance may stem from greater dependence on others for mobility and limited access to assistive technologies.^6^ Although Brazilian data suggest that schools present fewer barriers and more adaptations than other environments,^31^ and participants in this study reported to have appropriate assistive devices, accommodations may still be insufficient to prevent absenteeism. Transportation and financial constraints, lack of school staff training, and cultural beliefs about the educational potential of non‐ambulant individuals may further limit school participation. Future research should examine the impact of these factors on the school attendance of non‐ambulant individuals.

Brazilian adolescents and young adults with CP attend school less than children with CP. These findings are consistent with data from the Bangladesh CP Register, which also identified this age‐related factor as being associated with school attendance.^6^ In general, adolescents with CP tend to show lower participation not only in school but also in other areas of life.^32^ Several factors may contribute to reduced school attendance among Brazilian adolescents, including bullying and challenges related to transportation and mobility, particularly for non‐ambulant individuals.^33^, ^34^ Another important point to consider is that, according to Brazilian legislation, school attendance is mandatory only up to the age of 17 years. After this age, adolescents and young adults with poor academic outcomes may feel less motivated to continue their studies and may ultimately drop out. Efforts are highly necessary to promote the participation of adolescents and young adults with CP in school and in other areas of life.

### Limitations and directions for future studies

This study has some limitations worth noting. First, because of the structure of the BrCPR interviews, it was possible to capture mainly school attendance based on parent and caregiver reports, with no information on school involvement and real school frequency based on school reports. As aspects of involvement are crucial for understanding school inclusion, future studies should also explore the factors that influence the involvement of participation in school settings. Second, we were unable to assess whether participants had access to appropriate support or resources specifically related to school attendance. Access to assistive technology and adequately trained school staff may have an important role in supporting school participation. Third, we could not assess individuals' speech and communication limitations using more detailed tools, such as the Communication Function Classification System. Future studies should investigate how different levels of communication ability among individuals with CP are associated with school attendance. Finally, clinical features could be confirmed in health charts and by clinicians in only a few participants; most of the information was obtained solely through parent reports.

Data from our study are consistent with recently published Brazilian population‐based studies on CP, including the distribution of functional levels, clinical and socioeconomic characteristics, and the lower school attendance observed among individuals classified in higher GMFCS levels.^17^, ^35^ Therefore, although the data in the present study were collected between 2024 and 2025, we believe our findings are broadly representative of the Brazilian population with CP. Nevertheless, like other Brazilian population‐based studies, most data were obtained from the Southeast region, the wealthiest area of the country.^17^, ^35^ Thus, a substantial proportion of participants may have greater access to education and rehabilitation services. Given the marked socioeconomic inequalities across Brazilian regions, these findings may not fully reflect the realities of less affluent areas, particularly in the North region, where data on individuals with CP remain scarce. As the BrCPR expands geographically and the data collection period extends, future studies will be better positioned to provide a more comprehensive understanding of school attendance, especially regarding contextual and socioeconomic factors in low‐income regions of Brazil.

According to Brazilian law, school attendance is mandatory only up to 17 years of age. As mentioned in the previous sections, this may be one of the main reasons for school absenteeism in this age group. Because of the small proportion of adolescents and young adults in our sample, it was not possible to analyse them as a separate group and accurately evaluate the association between this age group and school attendance. Future studies including a larger sample of adolescents and young adults would allow a better analysis of this hypothesis.

Although the study shows adequate statistical power to detect differences in the analyses, some CIs remained wide and several *p*‐values were close to the significance threshold; therefore, caution must be taken when interpreting the results of this study. This is mainly because, despite the large overall sample size, several categories had small cell counts, which reduces the precision of the estimates. Additionally, the clinical and sociodemographic heterogeneity of participants increases variability in the adjusted model, leading to more conservative estimates. Therefore, the width of the CI reflects the uneven distribution of categories and the intrinsic variability of the sample rather than the absence of a true association. In addition, future analyses, including multilevel modelling with regional clustering, may help clarify contextual socioeconomic influences that were not detected in this initial model. This is particularly relevant in Brazil, where socioeconomic indicators are strongly patterned according to region.

### Conclusions

Rates of school attendance are higher in Brazil than in other LMICs. However, age, GMFCS level, and speech limitations are associated with school non‐attendance. Addressing speech support and accessibility is essential to improve school attendance.

## Supporting information


**Appendix S1:** Brazilian Cerebral Palsy Register form

## References

[1] Imms C , Adair B , Keen D , Ullenhag A , Rosenbaum P , Granlund M . “Participation”: A systematic review of language, definitions, and constructs used in intervention research with children with disabilities. Dev Med Child Neurol. 2016;58(1):29–38. 10.1111/dmcn.12932 26411643

[2] Shikako‐Thomas K , Lach L , Majnemer A , Nimigon J , Cameron K , Shevell M . Quality of life from the perspective of adolescents with cerebral palsy: “I just think I'm a normal kid, I just happen to have a disability.” Quality of Life Research. 2009;18(7):825–832. 10.1007/s11136-009-9501-3 19548117

[3] Souto DO , Cardoso S , Kelly F , Maciel DL . I Would Like to Do It Very Much ! Leisure Participation Patterns and Determinants of Brazilian Children and Adolescents With Physical Disabilities. 2023 Jul 1;35(3):304–312. 10.1097/PEP.0000000000001019 37095616

[4] Dan B , Rosenbaum P , Carr L , Gough M , Coughlan J , Nweke N . Updated description of cerebral palsy. Dev Med Child Neurol. 2026 Apr;68(4):465–476. 10.1111/dmcn.70149. 41612151

[5] United Nations . Sustainable Development Goals. United Nations Department of Economic and Social Affairs. https://sdgs.un.org/goals. Accessed November 27, 2025.

[6] Al Imam MH , Jahan I , Perrins G , Muhit M , Badawi N , Khandaker G . Predictors of school attendance among children with cerebral palsy in Bangladesh. Dev Med Child Neurol. 2025;67(5):665–675. 10.1111/dmcn.16136 39462437

[7] UNESCO . Global Education Monitoring Report 2020: Inclusion and Education – All Means All. Paris: UNESCO; 2020. Accessed June 11, 2025. https://unesdoc.unesco.org/ark:/48223/pf0000373718

[8] Mohammed FMS , Ali SM , Mustafa MAA . Quality of life of cerebral palsy patients and their caregivers: A cross sectional study in a rehabilitation center Khartoum‐Sudan (2014–2015). J Neurosci Rural Pract. 2016;07(03):355–361. 10.4103/0976-3147.182778 PMC4898102 27365951

[9] Sogbossi ES , Houekpetodji D , Kpadonou TG , Bleyenheuft Y . A Cross‐sectional Study of the Clinical Profile of Children With Cerebral Palsy in Benin, a West African Low‐Income Country. J Child Neurol. 2019;34(13):842–850. 10.1177/0883073819864516 31339409

[10] Andrews C , Kakooza‐Mwesige A , Almeida R , et al. Impairments, functional limitations, and access to services and education for children with cerebral palsy in Uganda: a population‐based study. Dev Med Child Neurol. 2020;62(4):454–462. 10.1111/dmcn.14401 31762018

[11] Nair Anish TS , Ramachandran R , Sivaram P , Mohandas S , Sasidharan A , Nair Sreelakshmi PR . Elementary school enrolment and its determinants among children with cerebral palsy in Thiruvananthapuram district, Kerala, India. J Neurosci Rural Pract. 2013;04(S 01):S40–S44. 10.4103/0976-3147.116446 PMC3808060 24174798

[12] Duke RE , Torty C , Okorie U , et al. Pattern of comorbidities in school‐aged children with cerebral palsy in Cross River State, Nigeria. BMC Pediatr. 2021; 21(1):165. 10.1186/s12887-021-02637-9 PMC8028192 33832457

[13] Alves A , Romão O , Da M , Capri R , Romão C . European Journal of Special Education Research Um Breve Relato Sobre A Educação Especial E Inclusiva No Brasil i. 5. 10.5281/zenodo.3726263

[14] Dutra C. A Política Nacional de Educação Especial na Perspectiva da Educação Inclusiva: um processo histórico de formulação e transformação. In: Desafios Da Educação Especial Na Perspectiva Inclusiva No Brasil. 2024:21–24.

[15] Brasil . Lei n° 13.146, de 6 de julho de 2015. Institui a Lei Brasileira de Inclusão da Pessoa com Deficiência (Estatuto da Pessoa com Deficiência) . Accessed June 11, 2025. https://www.planalto.gov.br/ccivil_03/_ato2015‐2018/2015/lei/l13146.htm

[16] IBGE . Censo 2022 . Accessed June 11, 2025. https://censo2022.ibge.gov.br/panorama/

[17] Chagas PSC , Lemos AG , Ayupe KMA , et al. Functioning profile and related impairments of children and adolescents with cerebral palsy ‐ PartiCipa Brazil preliminary results. BMC Pediatr. 2024;24(1):719. 10.1186/s12887-024-05210-2 PMC11552143 39529069

[18] Jahan I , Muhit M , Hardianto D , et al. Epidemiology of cerebral palsy in low‐ and middle‐income countries: preliminary findings from an international multi‐centre cerebral palsy register. Dev Med Child Neurol. 2021;63(11):1327–1336. 10.1111/dmcn.14926 34031872

[19] Ruiz Brunner M de las M , Jahan I , Cuestas E , et al. Latin American Cerebral Palsy Register (LATAM‐CPR): study protocol to develop a collaborative register with surveillance of children with cerebral palsy in Latin American countries. BMJ Open. 2023;13(12):e071315. 10.1136/bmjopen-2022-071315 PMC10729276 38070889

[20] Leite HR , da Silva LC , Magalhães ED , et al. Epidemiology of cerebral palsy in Brazil through the lens of the ICF framework. Dev Med Child Neurol. Published online 2026. 10.1111/dmcn.70222 41817275

[21] Rackauskaite G , Thorsen P , Uldall PV , Ostergaard JR . Reliability of GMFCS family report questionnaire. Disabil Rehabil. 2012;34(9):721–724. 10.3109/09638288.2011.615881 22011268

[22] Zuur AF , Ieno EN , Elphick CS . A protocol for data exploration to avoid common statistical problems. Methods Ecol Evol. 2010;1(1):3–14. 10.1111/j.2041-210X.2009.00001.x

[23] O'brien RM . A Caution Regarding Rules of Thumb for Variance Inflation Factors. Qual Quant. 2007;41(5):673–690. 10.1007/s11135-006-9018-6

[24] Smits DW , van Meeteren K , Klem M , Alsem M , Ketelaar M . Designing a tool to support patient and public involvement in research projects: the Involvement Matrix. Res Involv Engagem. 2020;6:30. 10.1186/s40900-020-00210-3 PMC7296703 32550002

[25] Ministry of Education . National Policy on Special Education from the Perspective of Inclusive Education (PNEEI) . Government of Brazil. Available at: https://www.gov.br/mec/pt‐br/pneei. Accessed December 2, 2025.

[26] President of the Republic . Statute of the Person with Disabilities (Law No. 13,146/2015) . Government of Brazil. Available at: https://www.planalto.gov.br/ccivil_03/_ato2015‐2018/2015/lei/l13146.htm. Accessed December 2, 2025.

[27] Coughlan J , Fitzgerald D . Enforcing the rights of persons with childhood‐onset disabilities: An international statement. Dev Med Child Neurol. 2025 Nov;67(11):1374–1375. 10.1111/dmcn.16458 40782359

[28] Cerebral Palsy Europe . Manifesto for the Rights of Persons With Cerebral Palsy. https://cerebralpalsyeurope.org/wp‐content/uploads/2024/09/manifesto‐English.pdf. Accessed December 1, 2025.

[29] Murphy A , Bailey B , Arciuli J . Exploring the Effects of Literacy Instruction for Children With Cerebral Palsy: A Systematic Review. Lang Speech Hear Serv Sch. 2023;54(1):299–321. 10.1044/2022_LSHSS-22-00014 36306504

[30] Carnevale LB , Berberian AP , Moraes PD de , Krüger S . Comunicação Alternativa no contexto educacional: conhecimento de professores. Revista Brasileira de Educação Especial. 2013;19(2):243–256. 10.1590/S1413-65382013000200008

[31] Alves ML de F , Souto DO , Romeros ACSF , et al. Characterization of environmental factors in children and adolescents with cerebral palsy in Minas Gerais: Participa Minas. Revista Paulista de Pediatria. 2024;42. 10.1590/1984-0462/2024/42/2023043 PMC10877669 38359317

[32] Javed U , Fuad M , Mahmood S , Rani B , Latif W . Evaluation of Participation Level of Adolescents with Cerebral Palsy. Pakistan Journal of Medical and Health Sciences. 2022;16(10):91–94. 10.53350/pjmhs22161091

[33] Pinquart M. Systematic Review: Bullying Involvement of Children With and Without Chronic Physical Illness and/or Physical/Sensory Disability—a Meta‐Analytic Comparison With Healthy/Nondisabled Peers. J Pediatr Psychol. Published online October 26, 2016:jsw081. 10.1093/jpepsy/jsw081 27784727

[34] Figueiredo PRP , Nóbrega RAA , Coster WJ , et al. Is Mobility Sufficient to Understand Community Participation of Adolescents and Young Adults With Cerebral Palsy? The Mediating and Moderating Roles of Contextual Factors. Arch Phys Med Rehabil. 2023;104(8):1227–1235. 10.1016/j.apmr.2022.12.195 36708858

[35] Borigato EVM , Dias BLS , Imamura EU , de Oliveira SB , Arci ECP , de Carvalho AL , da Silva APÁF , Marques FJP , Braga LW , et al. Cerebral palsy in Brazil: A multicentre, cross‐sectional, descriptive study. Dev Med Child Neurol. 2025;00:1–7. 10.1111/dmcn.70080 41345054

