# Supplementary material for: School attendance in individuals with cerebral palsy in Brazil
Source: Dev Med Child Neurol. 2026 May 21;68(12):1740–50. doi: 10.1111/dmcn.70316 (PMC13743679; doi:10.1111/dmcn.70316)
Supplement: Supplementary file 1 [file DMCN-68-1740-s001.pdf]

Supporting material 1: Data collection form

BRAZILIAN CEREBRAL PALSY REGISTRY FORM

The purpose of this form is to collect demographic and clinical data on individuals with Cerebral Palsy (CP). The data should be completed by a member of the RB-PC team involved in the care of the person with CP. **Part I** of the form includes information that must be gathered through an interview with the person with CP or their caregiver. **Part II** includes data on objective measures and classification systems and should be collected directly from the person with CP by the RB-PC team member completing the questionnaire. In this section, information provided by participants will also be highly valuable for accurate completion.

All RB-PC team members responsible for collecting the data in this form must watch all training videos provided by the RB-PC coordinator beforehand, particularly those addressing classification systems (GMFCS and MACS), muscle tone types, and anthropometry.

This form is not designed to be self-completed by the person with CP or their caregiver, but rather to be filled out in an interview format by the designated professional (a member of the RB-PC team). Finally, it is important not to leave any questions unanswered, except in cases where the information truly cannot be obtained.

Before beginning the interview, ask the caregiver or person with CP to have available any medical reports, evaluation summaries, and the child's health booklet. These documents may help with completing the questionnaire and improve data reliability. Also ensure that a measuring tape and scale are available for anthropometric records, along with the GMFM and MACS manuals.

The questions in this form are categorized according to the domains of the International Classification of Functioning, Disability and Health (ICF), as well as factors related to the etiology and risk of cerebral palsy (CP). The legend for the labels used is presented below:

- **ERF** Etiological and Risk Factors
- **BFS** Body Functions and Structures
- **AP** Activity and Participation
- **PF** Personal Factors
- **EF** Environmental Factors

Registro Brasileiro de Paralisia Cerebral (RB-PC)
Brazilian Cerebral Palsy Register
Global LMIC

PART I

INITIAL DATA

Form Data:

Date of form completion	Date
Institution where the form was completed	
Team member completing the form	
Email address of the team member completing the form	

Who is completing the form:

- ☐ Person with CP
- ☐ Caregiver of the person with CP
- ☐ Not applicable (form completed by the person with CP)

Contact Information of the Caregiver of the Person with CP

Name initials:

Relationship to the person with CP:

- ☐ Mother
- ☐ Father
- ☐ Sibling:
- ☐ Other:

Specify other:

Primary mobile number (including area code):
Secondary mobile number (including area code):
Email:

PF **Information on the Person with Cerebral Palsy (CP)**

Full name:
Name initials:
Date of birth (DD/MM/YYYY):

PF Sex at birth:

- ☐ Male
- ☐ Female

EF Living arrangement / Who does the person live with?

- ☐ Institutionalised
- ☐ With parents/caregivers
- ☐ Alone/shared housing/student residence/friend's house / their own home with partner, children
- ☐ Don't know

EF **If the person with CP lives with their family, please answer:**

Number of bedrooms in the household:	
Number of people living in the household	

EF Is the residence adapted to meet the needs of the person with CP?

- ☐ Yes
- ☐ No
- ☐ Partially
- ☐ Don't know
- ☐ Not applicable

EF **Residence**

State (Abbreviation)	
City	
Neighbourhood	
Postal code (ZIP)	
Primary mobile number:	
Secondary mobile number	

PF Study ID code for the person with CP (INICIAIS (INITIALS-SEX-DATE OF BIRTH-STATE ABBREVIATION. Example: FAG-F-22042007-SE): ________________________

EF **About the Family's Main Provider**
Who is the main provider of the household? *(Consider the main provider as the person who contributes the most financially to support the household.)*

- ☐ Person with CP
- ☐ Mother
- ☐ Father
- ☐ Grandparent
- ☐ Wife/husband
- ☐ Other:______________________________
- ☐ Don't know
- ☐ Not applicable

EF What is the education level of the family's main provider?

- ☐ No formal education or incomplete primary education
- ☐ Completed primary or incomplete secondary education
- ☐ Completed secondary or incomplete higher education
- ☐ Completed higher education (university) or postgraduate studies
- ☐ Don't know
- ☐ Not applicable

EF What is the employment status of the family's main provider?

- ☐ Business owner / Employer
- ☐ Formal employee (with work contract)
- ☐ Self-employed
- ☐ Unemployed
- ☐ No employment relationship (e.g., pensioner, retiree, receives governmental benefit)
- ☐ Public servant
- ☐ Other: ______________________________
- ☐ Don't know
- ☐ Not applicable

EF **What is the average total gross household income (in minimum wage equivalents)?**

- ☐ Less than 2 minimum wages
- ☐ 2 to 5
- ☐ 6 to 10
- ☐ 11 to 20
- ☐ More than 20
- ☐ Don't know
- ☐ Not applicable

Registro Brasileiro de Paralisia Cerebral (RB-PC)
Brazilian Cerebral Palsy Register
Global LMIC

BIRTH AND PREGNANCY INFORMATION
***If possible, ask the caregiver to have the child's health booklet available.**

ERF Place of Birth
- ☐ Hospital
- ☐ Emergency Care Unit
- ☐ Home birth
- ☐ Other: ____________________________
- ☐ Don't know

ERF If the birth was at home, was it planned?
- ☐ Yes
- ☐ No
- ☐ Not applicable
- ☐ Don't know

ERF Type of Delivery
- ☐ Spontaneous vaginal delivery
- ☐ Forceps-assisted delivery
- ☐ Planned caesarean section
- ☐ Unplanned/emergency caesarean section
- ☐ Other: ____________________
- ☐ Don't know

ERF Was there any complication or potential cause of CP during or before birth?
- ☐ Yes
- ☐ No
- ☐ Unknown

ERF **If yes,** when did the complication occur?
- ☐ Before and during delivery
- ☐ Only before delivery
- ☐ Only after delivery
- ☐ Don't know

ERF **If yes,** specify (check all that apply):
- ☐ Preeclampsia
- ☐ Eclampsia
- ☐ Maternal infection
- ☐ Hypoglycaemia
- ☐ Haemorrhage
- ☐ Premature rupture of membranes
- ☐ Prolonged labour
- ☐ Acute hypoxic event during delivery
- ☐ Others: ____________________________
- ☐ Don't know

ERF Gestational age:________________________

How gestational age was determined:
- ☐ Exact
- ☐ Approximate
- ☐ Not applicable

ERF **Birth-related data:**

Birth weight (grams)	
Birth length (cm)	
Head circumference at birth (cm)	
Apgar score	1':<br>5':

ERF How many prenatal visits were completed?
- ☐ Complete (4 or more visits)
- ☐ Incomplete (fewer than 4 visits)
- ☐ None
- ☐ Don't know

ERF Was there any sign of birth asphyxia (*e.g., very weak breathing, cyanosis, bradycardia, poor muscle tone, meconium aspiration, seizures*)?
- ☐ Yes
- ☐ No
- ☐ Unknown

If yes, specify:________________________________

ERF Did the child have any feeding difficulty during the first month of life, such as poor sucking ability?
- ☐ No
- ☐ Yes
- ☐ Unknown

ERF At birth, did the child require special care (*e.g., ventilatory support*)?
- ☐ Yes

- ☐ No
- ☐ Don't know

ERF **If yes**, was the child admitted to an intensive care unit (ICU) after birth?
- ☐ Yes
- ☐ No
- ☐ Don't know

ERF **If yes**, how many days was the child hospitalized in the ICU?
- ☐ Less than 1 month
- ☐ Between 1 and 3 months
- ☐ More than 3 months
- ☐ Don't know

ERF **If yes**, did the child need to be transferred to another specialized centre?
- ☐ No
- ☐ Yes –Which one? _________________

ERF Was a magnetic resonance imaging (MRI) scan performed?
- ☐ Yes
- ☐ No
- ☐ Don't know

ERF **If yes,** at what age was the MRI performed?
- ☐ Under 12 months
- ☐ Between 1 and 2 years
- ☐ 3 to 6 years
- ☐ 7 to 12 years
- ☐ 13 to 17 years
- ☐ Over 17 years
- ☐ Don't know

ERF Was this a multiple birth (*e.g., twins, triplets or more*)?
- ☐ Yes
- ☐ No
- ☐ Don't know

ERF **If yes**, specify:
- ☐ Twins
- ☐ Triplets
- ☐ 4
- ☐ 5
- ☐ 6
- ☐ More than 6

Birth order of the child with CP _______________

ERF Was fertility treatment used?
- ☐ Yes
- ☐ No
- ☐ Don't know

ERF **If yes,** please indicate the method used:
- ☐ Fertility medications
- ☐ Ovulation stimulation
- ☐ Artificial insemination
- ☐ Assisted reproductive technologies
- ☐ Other _____________________
- ☐ Don't know

ERF Number of previous live births (by the mother):__________

ERF Number of previous stillbirths (consider pregnancies at or beyond 20 weeks gestation): ___________

ERF Number of previous miscarriages (consider pregnancies that ended before 20 weeks of gestation): ___________

PARENT INFORMATION (BIOLOGICAL)

EF Was the person with CP adopted or under legal guardianship?

- ☐ Yes
- ☐ No
- ☐ Don't know

Considering different family structures (such as same-sex couples), it is important to explain to the interviewee, when applicable, that information about biological parents is being collected to understand potential genetic factors.

Is it possible to collect information about the biological parents?

- ☐ Yes
- ☐ No

ERF Were the biological parents living together at the time of birth?

- ☐ Yes
- ☐ No
- ☐ Don't know

Mother's Information

Name initials	
Date of birth	

ERF Mother's level of education at the time of the child's birth (with CP):

- ☐ Illiterate
- ☐ Completed primary education
- ☐ Incomplete primary education
- ☐ Completed secondary education
- ☐ Incomplete secondary education
- ☐ Completed higher education
- ☐ Incomplete higher education
- ☐ Completed postgraduate education
- ☐ Incomplete postgraduate education
- ☐ Don't know

ERF Mother's occupation at the time of the child's birth (with CP):

- ☐ Business owner / Employer
- ☐ An employee with a formal work contract
- ☐ Self-employed
- ☐ Unemployed
- ☐ Without formal employment (e.g., pensioner, retired, BPC recipient)
- ☐ Public servant
- ☐ Other________________________________
- ☐ Don't know

ERF How does the mother self-identify in terms of skin colour?

- ☐ Brown (Parda)
- ☐ White
- ☐ Black
- ☐ Indigenous
- ☐ Yellow
- ☐ Prefer not to answer / Don't know

Father's Information

Name initials	
Date of birth	

ERF Father's level of education at the time of the child's birth (with CP):

- ☐ Illiterate
- ☐ Completed primary education
- ☐ Incomplete primary education
- ☐ Completed secondary education
- ☐ Incomplete secondary education
- ☐ Completed higher education
- ☐ Incomplete higher education
- ☐ Completed postgraduate education
- ☐ Incomplete postgraduate education
- ☐ Don't know

ERF Father's occupation at the time of the child's birth (with CP):
- ☐ Business owner / Employer
- ☐ An employee with a formal work contract
- ☐ Self-employed
- ☐ Unemployed
- ☐ Without formal employment (e.g., pensioner, retired, BPC recipient)
- ☐ Public servant
- ☐ Other____________________________
- ☐ Don't know

ERF How does the father self-identify in terms of skin colour?
- ☐ Brown (Parda)
- ☐ White
- ☐ Black
- ☐ Indigenous
- ☐ Yellow
- ☐ Prefer not to answer / Don't know

ERF Are the parents blood relatives (e.g., first cousins, siblings)?
- ☐ Yes
- ☐ No
- ☐ Don't know

ERF Is there any other family member with a disability (parent, sibling, uncle/aunt of the person with CP)?
- ☐ Yes
- ☐ No
- ☐ Don't know

ERF **If yes**, what is this family member's relationship to the person with CP?
- ☐ Father
- ☐ Mother
- ☐ Sibling
- ☐ Grandparent
- ☐ Uncle/Aunt
- ☐ Other: ________________
- ☐ Don't know

ERF Specify the type of disability this family member has:
- ☐ Intellectual/mental (e.g., Down syndrome)
- ☐ Visual (e.g., blindness)
- ☐ Hearing (e.g., deafness)
- ☐ Motor (e.g., cerebral palsy)
- ☐ Communication/social interaction (e.g., Autism Spectrum Disorder)
- ☐ Don't know
- ☐ Other: ____________________

Comments (if there is more than one family member with a disability, specify here the relationship and type of disability of the second/third relative):

GENERAL HEALTH OF THE PERSON WITH CP

EF Does the person with CP have private health insurance or a private health plan?
- ☐ No
- ☐ Yes
- ☐ Don't know

EF Does the person with CP use the public health system (SUS)?
- ☐ No
- ☐ Yes
- ☐ Don't know

EF Are you aware of, or have you ever heard about, hip surveillance or the risk that people with CP may develop hip dislocation?
- ☐ No
- ☐ Yes
- ☐ Don't know

BFS Has the person with CP ever had a hip dislocation or subluxation?
- ☐ No
- ☐ Yes
- ☐ Don't know

EF **If yes**, has treatment for hip dislocation/subluxation been performed?
- ☐ No
- ☐ Yes
- ☐ Don't know

EF **If yes**, what type of treatment was performed?
- ☐ Soft tissue surgery in the groin (adductor tenotomy)
- ☐ Surgery with a screw in each hip (hemiepiphysiodesis)
- ☐ Surgery with plate and screws to relocate the hip (hip reconstruction)
- ☐ Surgery with plate and screws without relocating the hip (palliative surgery)
- ☐ Other
- ☐ Don't know

EF **If not,** why not?
- ☐ The caregivers chose not to proceed with surgery
- ☐ Medical condition not favourable for surgery
- ☐ Waiting for surgery through SUS
- ☐ Other: ______________________________
- ☐ Don't know

BFS Does the person with CP experience hip pain?
- ☐ Yes
- ☐ No
- ☐ Don't know

EF Has the person with CP ever had a hip X-ray?
- ☐ Yes
- ☐ No
- ☐ Don't know

EF At what age did the person with CP have their first pelvic/hip X-ray?
- ☐ <2 years
- ☐ 2–3 years
- ☐ 4–6 years
- ☐ 7–9 years
- ☐ >9 years

How often does the person with CP have hip X-rays?
- ☑ Every 6 months
- ☐ Once a year
- ☐ Once every two years
- ☐ Does not undergo regular X-rays
- ☐ Don't know

BFS Does the person with CP have difficulty swallowing?
- ☐ Yes
- ☐ No
- ☐ Don't know

BFS Does the person with CP have reflux?

- ☐ Yes
- ☐ No
- ☐ Don't know

AP Does the person with CP engage in physical activity?

- ☐ Yes, of light intensity
- ☐ Yes, of moderate to high intensity
- ☐ Yes, both light and moderate to high intensity
- ☐ No
- ☐ Don't know

CAUSE OR ETIOLOGY OF CP

*If you are unsure about any question, leave it blank.

ERF How many months or years old was the child when the caregiver or referring professional first noticed signs of developmental delay? (*consider chronological age*)

- ☐ 0–5 months
- ☐ 6–11 months
- ☐ 12 months or older
- ☐ Don't know

ERF Age at which CP was formally diagnosed for the first time

- ☐ < 6 months
- ☐ 6–12 months
- ☐ 13–23 months
- ☐ 2–5 years
- ☐ 6–9 years
- ☐ > 10 years
- ☐ Don't know

ERF Which medical professional made the diagnosis?

- ☐ Paediatrician
- ☐ Neurologist
- ☐ Physiatrist
- ☐ Other: _______________
- ☐ Don't know

ERF When did the cause of CP occur?

- ☐ During pregnancy and up to the first 28 days of life (pre- and perinatal)
- ☐ After the first 28 days of life (postnatal)
- ☐ Unknown
- ☐ Don't know

ERF <u>Cause: If confirmed during pregnancy and up to the first 28 days of life (pre- and perinatal)</u>

- ☐ Unknown
- ☐ Cytomegalovirus in utero
- ☐ Infections (toxoplasmosis, rubella, herpes simplex virus)
- ☐ Zika virus
- ☐ Brain malformation
- ☐ Jaundice
- ☐ Periventricular haemorrhage or leukomalacia
- ☐ Perinatal hypoxic-ischaemic event
- ☐ Other infections: ____________________
- ☐ Other causes: ______________________
- ☐ Don't know

ERF <u>If after the first 28 days of life (postnatal)</u>

Head trauma

- ☐ Car accident
- ☐ Fall
- ☐ Other: ______________________
- ☐ Not applicable
- ☐ Don't know

Infection:

- ☐ Cause not specified
- ☐ Viral
- ☐ Bacterial
- ☐ Dehydration due to gastroenteritis
- ☐ Not applicable
- ☐ Don't know

Heart attack or stroke:
- ☐ Surgical procedure
- ☐ Spontaneous
- ☐ Associated with other cardiac complications
- ☐ Not applicable
- ☐ Don't know

Other causes:
- ☐ Post-seizure
- ☐ Near-miss sudden infant death syndrome
- ☐ Post-immunisation
- ☐ Submersion/drowning
- ☐ Perioperative hypoxia
- ☐ Apparent Life-Threatening Event (ALTE)
- ☐ Don't know
- ☐ Not applicable

REHABILITATION

EF Has the person with CP ever received any rehabilitation therapy or other related support at any point in life?
- ☐ Yes
- ☐ No
- ☐ Don't know

EF **If yes,** please indicate which service(s)/support you received (you may select more than one):
- ☐ Physiotherapy
- ☐ Speech therapy
- ☐ Occupational therapy
- ☐ Psychology
- ☐ Special school
- ☐ Home-based assistance (home visits)
- ☐ Transportation
- ☐ Other: ______________________________
- ☐ Don't know

EF Is the person with CP currently receiving rehabilitation services?
- ☐ Yes
- ☐ No
- ☐ Sometimes
- ☐ Don't know

EF **If you answered "no" or "sometimes,"** what is the reason?
- ☐ Lack of specialised professionals or human resources
- ☐ Recurrent illness
- ☐ No rehabilitation centre in the area
- ☐ Lack of transportation
- ☐ Not applicable
- ☐ Other: ____________
- ☐ Don't know

EF How many hours per week does the person with CP receive rehabilitation?
- ☐ Less than 1 hour
- ☐ 1 to 3 hours
- ☐ 4 to 7 hours
- ☐ 8 to 11 hours
- ☐ More than 11 hours
- ☐ Don't know

EF Are all therapies provided at the same centre/place?
- ☐ Yes
- ☐ No
- ☐ Don't know

EF Does the person with CP receive all the medical and rehabilitation services they need?
- ☐ Yes
- ☐ No
- ☐ Don't know

EF **If no,** which service(s) do you think are missing?
- ☐ Orthopaedics
- ☐ Neurology
- ☐ Ophthalmology
- ☐ Psychiatry
- ☐ Physiotherapy
- ☐ Occupational therapy

☐ Speech therapy
☐ Visual stimulation
☐ Psychology
☐ Educational psychology
☐ Other: ____________________
☐ Don't know

EF What type of centre provides the rehabilitation services?

☐ Public
☐ Private
☐ Public/Private
☐ Not applicable
☐ Don't know

EF At what age did the person with CP first receive rehabilitation services?

☐ Over 6 months
☐ 6 to 12 months
☐ 1 year
☐ 2 years
☐ 3 years
☐ 4 years
☐ 5 years
☐ 7 years
☐ 8 years
☐ 9 years
☐ Over 10 years
☐ Not applicable
☐ Don't know

EF Does the person with CP have all the assistive technologies/equipment prescribed by a doctor or therapist?

☐ Yes
☐ No
☑ Partially
☐ No assistive/adaptive equipment needed
☐ Don't know

EF Are there adaptations in the home that support daily living activities for the person with CP?

☐ Yes
☐ No
☐ Partially
☐ Don't know

GOVERNMENTAL DISABILITY BENEFIT (*BPC*)

EF Does the person with CP receive the Disability Benefit (*BPC – Benefício de Prestação Continuada*)?

☐ Yes
☐ No
☐ Don't know

EF At what age did the person with CP begin receiving the BPC benefit? (Please specify in years or months):__________

EF Does the person with CP receive a disability pension?

☐ Yes
☐ No
☐ Don't know

EF Does the person with CP receive any other type of pension besides BPC?

☐ Yes
☐ ☐ No
☐ ☐ Don't know

If yes, please specify: ________________

OCCUPATION OF THE PERSON WITH CP

AP Does the person with CP currently attend or has ever attended school?

☐ Yes, currently attending
☐ Attended in the past, but not currently

☐ 6 years

☐ Not yet at school age
☐ Has never attended school
☐ Don't know

PF Is the person with CP fully literate (*i.e., can read and write*)?

- ☐ Yes
- ☐ No
- ☐ Not applicable
- ☐ Don't know

PF What is the highest level of education attained?

- ☐ Completed primary education
- ☐ Incomplete primary education
- ☐ Completed secondary education
- ☐ Incomplete secondary education
- ☐ Completed higher education
- ☐ Incomplete higher education
- ☐ Completed postgraduate/specialization
- ☐ Incomplete postgraduate/specialization
- ☐ Completed master's degree
- ☐ Incomplete master's degree
- ☐ Completed doctorate
- ☐ Incomplete doctorate
- ☐ Not applicable / not of school age

AP Does the person with CP currently have a paid job or employment?

- ☐ Yes
- ☐ No
- ☐ Not of working age
- ☐ Don't know

If not, is the person currently seeking employment?

- ☐ Yes
- ☐ No
- ☐ Don't know

Registro Brasileiro de Paralisia Cerebral (RB-PC)
Brazilian Cerebral Palsy Register
Global LMIC

Part II

CLINICAL DATA OF THE PERSON WITH CP

BFS CP Classification (ICD-10): Select one option. If mixed, select two options.

- ☐ Spastic CP
- ☐ Dyskinetic CP (dystonic/choreoathetotic)
- ☐ Ataxic CP
- ☐ Hypotonic
- ☐ Don't know

BFS **If you selected spastic**, what is the subtype?

- ☐ Spastic quadriplegic CP
- ☐ Spastic diplegic CP
- ☐ Spastic hemiplegic CP
- ☐ Not applicable / not spastic
- ☐ Don't know

CEREBRAL PALSY CLASSIFICATION SYSTEM

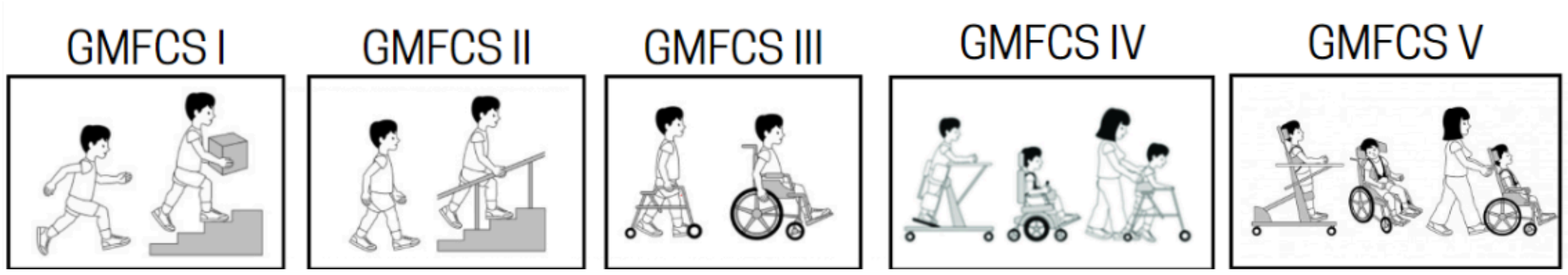

AP **GMFCS**

- ☐ Level I (walks without assistance on all surfaces)
- ☐ Level II (walks without assistance, but uses a handrail for stairs)
- ☐ Level III (uses a mobility aid to walk and may use a wheelchair for long distances)
- ☐ Level IV (sits independently, but needs significant support to stand or walk)
- ☐ Level V (has difficulty sitting independently and controlling head and body posture)
- ☐ Unable to classify/don't know

How was the GMFCS level obtained?

- ☐ Medical report or written assessment
- ☐ Questionnaire or family report
- ☐ Observed by the data collector
- ☐ Reported by the person with CP or their caregiver
- ☐ Unable to obtain

AP **Mini-MACS (1- 4 years) or MACS (4-18 years)**

- ☐ Level I (handles objects easily and successfully)
- ☐ Level II (handles most objects but with reduced quality and/or speed)
- ☐ Level III (handles objects with difficulty; needs help to prepare and/or modify activities)
- ☐ Level IV (handles a limited selection of easily managed objects; always requires assistance)
- ☐ Level V (does not handle objects and has severely limited ability to perform even simple hand actions)
- ☐ Unable to classify/don't know

Como o Sistema de Classificação da Habilidade Manual (MACS) foi obtido?

- ☐ Medical report or written assessment
- ☐ Questionnaire or family report
- ☐ Observed by the data collector
- ☐ Reported by the person with CP or their caregiver
- ☐ Unable to obtain

Registro Brasileiro de Paralisia Cerebral (RB-PC)
Brazilian Cerebral Palsy Register

Global LMIC Cerebral Palsy Register

CONGENITAL ALTERATIONS

ERF Were there any conditions present at birth? (e.g., congenital heart defect)

☐ No
☐ Yes
☐ Don't know

If yes, please specify: ________________

ERF Is there any congenital syndrome associated with CP?

☐ No
☐ Yes
☐ Don't know

If yes, please specify: _____________________

PRESENCE OF ASSOCIATED CONDITIONS

Mark one option for each section

BFS **Epilepsy:**

☐ Never had
☐ Yes, and uses medication
☐ Had one or few seizures and does not use medication
☐ Don't know

BFS **Intellectual/Cognitive:**

☐ No alterations, general population (IQ > 69)
☐ Mild (IQ 50–69)
☐ Moderate (IQ 35–49)
☐ Severe (IQ < 35)
☐ Yes, but the degree (IQ) is unknown
☐ Don't know / child too young

BFS **Visual:**

☐ No alterations
☐ Low vision (corrected with glasses)
☐ Low vision (uncorrected with glasses)
☐ Blindness
☐ Don't know / child too young

BFS **Strabismus:**

☐ Yes
☐ No
☐ Don't know

BFS **Hearing:**

☐ No alterations
☐ There is some hearing impairment
☐ Bilateral hearing loss (hypoacusis) / complete deafness
☐ Don't know / child too young

1	2	3	4	5	6	7	8	9	10

For the next 4 items, please use the scale to rate the level of difficulty related to each corresponding item, based on the perception of the caregiver or the person with CP. If it is not possible to obtain this information this way, please answer based on your knowledge about the person with CP.

"Considering a scale from 1 to 10, where 1 means little difficulty and 10 means extremely difficult, what score would you give for:"

AP **Speech/verbalization:**

☐ Speech without limitations
☐ 1 to 3 (mild limitation)
☐ 4 to 7 (moderate limitation)
☐ 8 to 10 (severe limitation)
☐ Don't know/child too young

AP Difficulty eating or drinking independently:

- ☐ Eats and drinks independently with safety and efficiency
- ☐ 1 to 3 (mild limitation)
- ☐ 4 to 7 (moderate limitation)
- ☐ 8 to 10 (severe limitation)
- ☐ Don't know/child too young

AP Does the person with CP have difficulty bathing independently?

- ☐ No difficulty
- ☐ 1 to 3 (mild limitation)
- ☐ 4 to 7 (moderate limitation)
- ☐ 8 to 10 (severe limitation)
- ☐ Don't know/child too young

AP Does the person with CP have difficulty dressing independently?

- ☐ No difficulty
- ☐ 1 to 3 (mild limitation)
- ☐ 4 to 7 (moderate limitation)
- ☐ 8 to 10 (severe limitation)
- ☐ Don't know/child too young

ERF Does the person with CP have any associated/ secondary diagnoses (e.g., Autism Spectrum Disorder, Down syndrome, among others)?

- ☐ Yes
- ☐ No
- ☐ I don't know

If yes, please specify: ____________________

ANTHROPOMETRY

**At the time of completing this form*

BFS Anthropometric measures:

Current weight (kg):	
Height (cm):<br>(Do not collect if the person has lower limb contractures)	

Who provided this information?

- ☐ Professional
- ☐ Person with CP/caregiver

How was the height measured by the professional?

- ☐ Standing
- ☐ Lying down
- ☐ Measurement not possible

BFS Arm circumference and heel-to-knee distance

arm circumference (Mark the midpoint between the acromion and the olecranon, and measure the circumference at this point):	
Lateral distance from heel to knee (Always measure on the **left** side. If this side is more affected, measure on the right side. Measure from the lateral base of the calcaneus to the midpoint of the base of the patella)	

If you, the professional or the participant, would like to leave a comment, please use the space below:

